# European Hedgehogs as Hosts of Chaphamaparvovirus, Italy

**DOI:** 10.3390/ani14243624

**Published:** 2024-12-16

**Authors:** Federica Di Profio, Barbara Di Martino, Gianvito Lanave, Serena Robetto, Ilaria Prandi, Maria Teresa Capucchio, Maria Lucia Mandola, Giuseppe Quaranta, Riccardo Orusa, Fulvio Marsilio, Vito Martella, Vittorio Sarchese

**Affiliations:** 1 Department of Veterinary Medicine, Università degli Studi di Teramo, Località Piano D’Accio, 64100 Teramo, Italy; bdimartino@unite.it (B.D.M.); fmarsilio@unite.it (F.M.); vsarchese@unite.it (V.S.); 2 Department of Veterinary Medicine, Università Aldo Moro di Bari, S.p. per Casamassima Km3, 70010 Bari, Italy; gianvito.lanave@uniba.it (G.L.); vito.martella@uniba.it (V.M.); 3 Centro di Referenza Nazionale per le Malattie degli Animali Selvatici (CeRMAS), Istituto Zooprofilattico Sperimentale del Piemonte, della Liguria e della Valle d’Aosta, 11020 Aosta, Italy; serena.robetto@izsto.it (S.R.); riccardo.orusa@izsto.it (R.O.); 4 Centro Animali Non Convenzionali (C.A.N.C), Department of Veterinary Sciences, University of Turin, 10095 Turin, Italy; ilaria.prandi@unito.it (I.P.); mariateresa.capucchio@unito.it (M.T.C.); giuseppe.quaranta@unito.it (G.Q.); 5 S.S. Virologia Specialistica, Istituto Zooprofilattico Sperimentale Piemonte, Liguria e Valle d’Aosta, 10154 Turin, Italy; marialucia.mandola@izsto.it; 6 Department of Pharmacology and Toxicology, University of Veterinary Medicine, 1078 Budapest, Hungary

**Keywords:** novel parvovirus, HhChPV, *Erinaceus europaeus*

## Abstract

In 2022, a novel parvovirus was identified from an outbreak of fatal enteritis in weaned European hedgehogs (*Erinaceus europaeus*) at a wildlife rescue center in Southern Italy. During sequence analysis, the strain was found to be closely related (90.4% nucleotide identity) to a chaphamaparvovirus (ChPV) discovered in Amur hedgehogs (*Erinaceus amurensis*) during a large metaviromic investigation in game animals in China. In this study, we investigated the presence of this novel ChPV in necropsied European hedgehogs from different areas of North-Western Italy. Duodenal and liver samples collected from 194 necropsied hedgehogs were screened by using a specific quantitative PCR. A total of 38/194 animals (19.6%) tested positive, with ChPV DNA being detected in the duodenum (9.3%, 18/194), liver (7.2%, 14/194) or in both (3.1%, 6/194) tissue samples, with comparable rates and mean viral loads. The nearly full-length genome of four hedgehog ChPV strains was reconstructed. During phylogenetic analysis based on the NS1 and partial VP aa sequences, the four strains detected in this study tightly clustered with the prototype ChPVs previously identified in Amur and European hedgehogs within a potential novel candidate species of the genus *Chaphamaparvovirus*.

## Simple Summary

Recently, during an outbreak of fatal enteritis involving European hedgehogs housed in a wildlife rescue center in Apulia Region (Southern Italy), a novel parvovirus closely related to chaphamaparvoviruses was identified. In this study, by using hedgehog chaphamaparvovirus (HhChPV)-specific primers and a probe, viral DNA was detected in duodenal and liver samples collected from necropsied European hedgehogs obtained from different areas of North-Western Italy, with an overall prevalence of 19.6% (38/194). When assessing the nearly complete genomes of four HhChPVs, the identified strains were genetically highly related (89.7–97.7% nucleotide identity) to the HhChPVs previously found in Amur and European hedgehogs. Upon phylogenetic analysis, all the Italian and Chinese HhChPV strains were tightly clustered as members of a proposed novel species in the genus *Chaphamaparvovirus*. Molecularly investigating the hedgehog virome is crucial for understanding the roles of these animals in the ecology of viral pathogens, which may pose threats to vulnerable hedgehog populations, and from a One Health perspective, given the synanthropic behavior of hedgehogs, for providing valuable insights into potential zoonotic risks.

## 1. Introduction

Parvoviruses are structurally simple viruses with linear single-stranded DNA genomes and nonenveloped icosahedral capsids, able to infect a wide range of animals, from insects to humans [1]. In recent years, novel parvoviruses have been described due to advances in sequencing techniques and metagenomic analyses, leading to recent taxonomical re-classification characterized by the introduction of the novel subfamily *Hamaparvovirinae* [2]. Within this family, members of the genus *Chaphamaparvovirus* have been identified in several animal species, including bats, rodents, birds, pigs, and pets [3,4,5,6,7,8,9,10,11,12]. Chaphamarvoviruses (ChPVs) have been associated with a variety of clinical signs in some animal hosts. The mouse kidney parvovirus (MKPV) (*Chaphamaparvovirus rodent 1* species) was previously recognized as the cause of inclusion body nephropathy (IBN) and kidney fibrosis in mice [9,13]. The possible pathogenetic role of ChPV was also suspected in a dead peafowl with enteritis and pneumonia [14], in bearded dragons showing respiratory or neurological symptoms [15], and in dogs and cats with acute gastroenteritis and upper respiratory tract disease [11,12,16]. The European hedgehog (*Erinaceus europaeus*; Linnaeus, 1758) is a small, nocturnal insectivore (order *Eulipotyphla*) widely distributed in Europe [17]. The ecological versatility of these animals allows them to thrive in diverse habitats, including wild and urban environments. The synanthropic attitudes result in frequent contacts with sympatric wild and domestic species, including humans, raising the possibility of their involvement as carriers and/or hosts of several potentially emerging and zoonotic viruses [18,19,20]. In 2022, a new candidate ChPV species was detected in orphaned weaned European hedgehogs, housed in the Regional Wildlife Rescue Centre of Bitetto (prefecture of Bari, Apulia Region, Italy), where increased mortality associated with enteritis was observed during the period June–July 2022. By conducting a metaviromic investigation, ChPV sequences were identified in pooled stool samples of three hedgehogs, and by qPCR, ChPV DNA was detected in the gastrointestinal tracts of an additional nine deceased animals, all of which showed a similar cohort of clinical signs, such as the production of semi-solid, dark red, fetid feces and, in some cases, respiratory disease with sneezing and mild serous nasal discharge [21]. During sequence analysis, the novel hedgehog ChPV (HhChPV, strain ITA/2022/hedgehog/265) was closely related (90.4% nucleotide [nt] identity) to a ChPV strain (HeN-F2) identified in the pooled fecal samples of 11 healthy Amur hedgehogs (*Erinaceus amurensis;* Schrenk, 1858) during a large metaviromic investigation conducted in game animals in China [22]. However, based on the limited literature, it remains unclear whether this virus is common in hedgehogs or if it has only been sporadically detected. Herein, to address this gap and better understand the epidemiology of HhChPV in these small mammals, we screened a large collection of samples obtained from European hedgehogs from different areas of North-Western Italy.

## 2. Materials and Methods

### 2.1. Sampling

Molecular screening for HhChPV was performed on tissue samples (duodenum and liver) collected from a total of 194 necropsied European hedgehogs between March 2018 and December 2022. Animals were identified by their external morphology, including their distinctive short, grooved spines covering the entire dorsum of the body and fairly small eyes [23,24]. Out of 194, 146 animals were admitted to “Centro Animali Non Convenzionali (C.A.N.C.)” of the Department of Veterinary Sciences of Turin University (collection A), and 37 additional animals were hospitalized at a specialized center for treatment and rehabilitation of European hedgehogs “La Ninna” (collection B), located in Cuneo prefecture. Paired duodenal and liver specimens were sampled by the Istituto Zooprofilattico Sperimentale Piemonte, Liguria e Valle d’Aosta, from additional 11 hedgehog carcasses (collection C) retrieved in north-western regions of Italy between February and September 2019, following the framework of a national passive surveillance program. When available, information about the sex, age (categorized as unweaned, juvenile, and adult according to external characteristics), date of admission, date of death, and cause of death (trauma/predation, neoplasia, infectious/parasitic diseases, starvation, unknown) of each animal was recorded. All tissues were frozen and transported to the Department of Veterinary Medicine (University of Teramo, Italy) for virological investigations.

### 2.2. Screening of Samples in Quantitative and Conventional PCR

Tissue samples (1 g each), homogenized and processed as previously described [25], were subjected to nucleic acid extraction using TRIzol LS (Invitrogen, Ltd., Paisley, UK). The presence of HhChPV DNA was assessed by specific real-time PCR (qPCR), targeting a 119 nt segment of the nonstructural protein 1 (NS1) encoding gene [21]. Quantification was performed using TaqMan Fast Advanced Master Mix (Invitrogen Ltd., Milan, Italy) in a 25 μL mixture comprising 5 μL of extracted DNA and 20 μL of master mix. The primers Chap ErEu/2406-F, Chap ErEu/2407-R, and probe Chap ErEu/316-Pb (Table 1) were used at concentrations of 200 and 100 nM, respectively. Thermal cycling consisted of 42 cycles of denaturation at 95 °C for 10 s and annealing-extension at 60 °C for 30 s. HhChPV DNA copy numbers were determined based on standard curves generated by 10-fold dilutions of a plasmid standard TOPO XL PCR (ThermoFisher Scientific, Waltham, MA, USA) containing a 500 nt fragment of the NS1 region of the strain ITA/2022/hedgehog/265 (GenBank accession no. OQ919797) [21]. All the qPCR positive samples were re-tested in qualitative PCR using specific HhChPV primers, pair 2421_HhChPV F and 2410_HhChPV R (Table 1), designed to amplify a 1029 nt region of the NS1 gene [21].

### 2.3. Genome Sequencing and Phylogenetic Analysis

The PCR products were purified using a QIAquick gel extraction kit (Qiagen GmbH, Hilden, Germany) and subjected to direct Sanger sequencing using BigDye Terminator Cycle chemistry (Applied Biosystems, Foster City, CA, USA). The Basic Local Alignment Search Tool (BLAST; http://www.ncbi.nlm.nih.gov, accessed on 9 September 2024) and FASTA (http://www.ebi.ac.uk/fasta33, accessed on 9 September 2024) with default values were used to find homologous hits. In addition, positive samples with viral loads > 10^4^ copies/mL of template were selected for attempts to amplify complete genomes by using primer pairs designed on the consensus sequences of the two HhChPV genomes recovered from the NCBI database (Table 1) [21]. Long PCRs were performed with TaKaRa La Taq polymerase (Takara Bio Europe S.A.S., Saint-Germain-en-Laye, France). Amplicons were purified and cloned using a TOPO XL Cloning Kit (ThermoFisher Scientific, Waltham, MA, USA), sequencing at least three clones for each PCR to generate consensus sequences. Open reading frame (ORF) predictions and annotations were performed using Geneious Prime software Version 2022.2.2 (Biomatters Ltd., Auckland, New Zealand). Multiple sequence alignments were generated using MAFFT [26]. The phylogenetic analyses were performed using the Neighbor-Joining method implemented using MEGA 11 version 10.0.5 software [27].

## 3. Results

Using HhChPV-specific primers and probe [21], viral DNA was detected in 38/194 hedgehogs (19.6%). HhChPV DNA was detected in 25/146 (17.1%) hedgehogs from collection A, in 11/37 (29.7%) animals from collection B, and in 2/11 (18.2%) animals belonging to collection C. All positive hedgehogs of A and B collections were from Piedmont Region, while animals of collection C were sampled in Valle D’Aosta Region. Additional details are summarized in Table S1. Specifically, out of 38 infected hedgehogs, 24 (63.2%) were male and 14 (36.8%) were female. Furthermore, 26 hedgehogs (68.4%) were classified as adult and 12 (31.6%) as juvenile. Most positive cases were admitted in spring months (31.6%, 12/38), followed by summer months (28.9%, 11/38) and autumn months (23.7%, 9/38), while only one animal (2.6%, 1/38) was admitted in February, and data were not available for 5/38 cases. Regarding the timing of death, 14 hedgehogs died on the day of admission, 11 died between 1 and 5 days later, and 4 died after 21, 23, 30, and 60 days, respectively. Concerning the causes of death, a high number of animals (55.3%, 21/38) died due to traumatic lesions and 14 (36.8%, 14/38) due to infectious or parasitic diseases, while the cause of death remained unknown for 3 hedgehogs.

Viral DNA was identified either in intestinal or liver samples or in both with rates of 9.3% (18/194), 7.2% (14/194), and 3.1% (6/194), respectively. The viral loads ranged from 7.87 × 10^2^ to 3.47 × 10^5^ DNA copies/g (mean 6.43 × 10^4^ DNA copies/g) in the intestinal contents and from 5.93 × 10^2^ to 2.67 × 10^6^ DNA copies/g (mean 2.37 × 10^5^ DNA copies/g) in liver specimens. For eight virus-positive necropsied animals, it was possible to screen additional internal organs, revealing the presence of viral DNA in the kidneys (7/8), spleen (3/8), and lungs (1/8), with the highest viral loads found in kidneys with titers ranging from 4.67 × 10^2^ to 1.21 × 10^8^ DNA copies/g (mean 2.18 × 10^7^ DNA copies/g) (Table 2).

The partial NS1 gene sequences, 1029 nt in length, of 11 HhChPV strains (GenBank accession no. PQ112546-PQ112556) and the nearly complete genome sequences of 4 HhChPV strains were generated. The sequenced strains were detected in hepatic (strains 7L/2019/ITA, 1123L/2019/ITA, PQ112557, and PQ112559), duodenal (strains 1279DU/2019/ITA and PQ112560), and renal (strains 637K/2022/ITA and PQ112558) tissues. For the whole genome sequencing of four strains, a contiguous sequence ranging between 3583 and 3597 nt was obtained, with an overall nt identity of 98.7–99.4% to each other and 89.7–97.7% to the two HhChPV strains available to date in the GenBank database [21,22]. The genome features of the identified strains comprised two ORFs, encoding for the NS1 protein (668 aa), and a partial VP protein (392–397 aa). An additional ORF, predicted to encode a small accessory protein p15 (137 aa), partially overlapped the N-terminal of the NS1 ORF. As observed in all amniote ChPVs [28], a partial ORF encoding a spliced NS2 protein was identified. The coding sequence for this protein started at nt 1 and ended at nt 2351, with an intron region from nt 68 (donor site: AG¯GT) to nt 1704 (acceptor site: CA¯G). This resulted in a 237 amino acid (aa) partial NS2 protein, while the non-spliced variant of NS2 (nt 1683–2351) was 222 aa in length. The NS1 genes of the four Italian strains were characterized by the putative start codon MQA located in an adequate Kozak consensus sequence (**A**CAATGC) [29] and contained two conserved replication initiator (endonuclease) motifs: ^95^I**H**V**H**LLAL^102^ (the boldface type indicates conserved amino acids) and ^149^SLLA**Y**MA **K**^156^ [30]. Moreover, the highly conserved helicase domain Walker motifs, including Walker A (^312^**GP**TNT**GK**S^319^), B (^350^IGIW**EE**^355^), B′ (^367^**K**QIFE**G**METSIPV **K**^380^), and C (^392^IFI**T**T**N**^397^), were identified in the NS1 [31,32]. The termination of the NS1 ORF overlapped the start of the capsid ORF by 8 nt. As observed for other members of the subfamily *Hamaparvovirinae* [2], the poly-glycine and conserved phospholipase A2 motifs, HDXXY and YXGXG [33], were absent in the VP proteins of all HhChPVs, including the strains detected in this study. In addition, similar to other ChPVs, the first methionine of the VP ORF was preceded by a potential coding sequence, and a canonical splice acceptor site (CA¯G) was located directly upstream [2].

**Figure 1:**
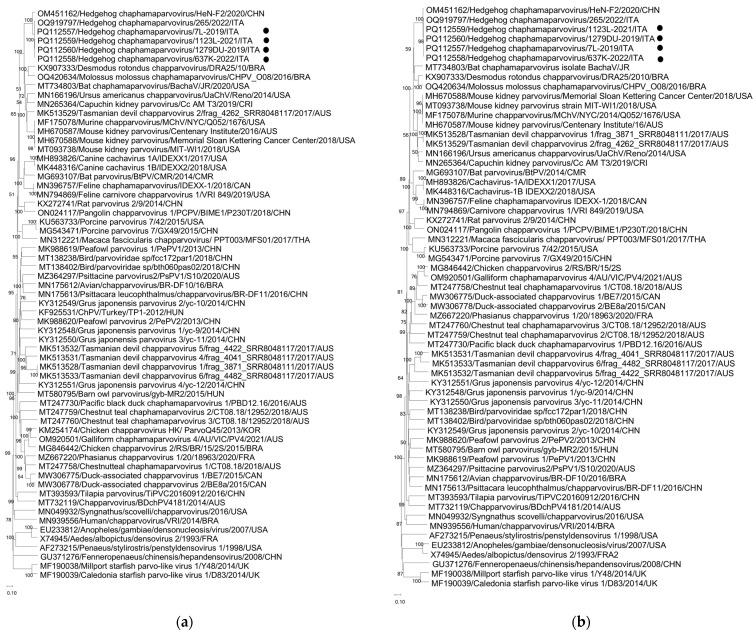
Phylogenetic analyses based on the NS1 (**a**) and partial VP (**b**) aa sequences of the HhChPVs identified in this study. The trees, constructed with a selection of ChPV strains representative of each species, were generated using the Neighbor-Joining method based on the p-distance correction and supplying statistical support with the bootstrapping of 1000 replicates. Bootstrap values > 50% are shown. Labels indicate the HhChPV strains detected in this study. Evolutionary analyses were conducted using MEGA 11.

For the phylogenetic analysis of the complete NS1 amino acid sequences (Figure 1a), the four HhChPVs detected in this study tightly segregated (bootstrap value 100%) with the Italian strain ITA/2022/hedgehog/265 and with the Chinese strain HeN-F2 (aa identity of 91.5–99.0%) in a well-defined group including ChPVs identified in bats, rodents, monkeys, bears, and Tasmania devils, with the closest relatives represented by parvoviruses from bats (60.3–62.5%) [8,34]. A similar clustering pattern was confirmed in the partial VP aa sequence-based phylogenetic tree (Figure 1b), in which the HhChPVs were genetically more related to bat ChPVs (68.2–70.5%) [8,34,35].

## 4. Discussion

In this study, we extended the research of HhChPV to tissue samples of 194 European hedgehogs, mostly (*n* = 183) obtained from two wildlife rescue centers located in North-Western Italy. The novel parvovirus was detected at a high prevalence rate (19.6%, 38/194). HhChPV DNA was found in the duodenum and/or liver tissues with comparable rates (12.4% vs. 10.3%) and mean viral loads (2.37 × 10^5^ vs. 6.43 × 10^4^ DNA copies/g). Notably, the tropism of ChPV for the intestinal tract has already been reported in other animal species, including dogs [11,36] and cats [12,16], and these viruses have also been found in the liver of an American black bear [37] and found to be associated with hepatitis in pheasants [38] and chickens [39].

In our analysis, HhChPV was also detected in the internal organs of eight hedgehogs, including kidneys and/or spleen and lungs. Together, these findings could be accounted for by systemic infection with the hematogenous spreading of the virus, consistent with the previous report identifying viral DNA in the stools, liver, kidneys, and spleen of hedgehogs with fatal enteritis [21].

Interestingly, in our study, by qPCR (Table 2), while HhChPV DNA was detected in the duodenum (6/8, 75.0%), liver (2/8, 25.0%), spleen (3/8, 37.5%), and lungs (1/8, 12.5%) with lower rates, viral DNA was found in nearly all animals in the kidneys (7/8, 87.5%), with the overall highest viral loads (1.21 × 10^8^ and 3.13 × 10^7^ DNA copies/g) found in two hedgehogs. This trend was also observed in the other HhChPV-infected hedgehogs, likely hinting at a preferential tropism of HhChPV for kidneys. In bats [8,28,34] and non-human primates [28], ChPVs have also been found in the kidneys. Even more interestingly, mouse kidney parvovirus (MKPV) is kidney-tropic [9,28,40], thus leading to the hypothesis that many mammal chaphamaparvoviruses are nephro-tropic [28]. It is worth noting that for MKPV, a clear causal association with clinical signs or lesions has been demonstrated, fulfilling the Fredrichs and Rellman criteria. MKPV is indeed associated with severe chronic interstitial nephropathy and renal failure in immunocompromised mice [9,28].

Concerning the hedgehogs involved in this investigation, traumatic lesions were identified as the primary cause of death in 21 subjects, infectious or parasitic diseases were suspected in 14 cases, and the cause of death remained unknown for 3 hedgehogs. Data about histopathological examination were available only for 23 necropsied positive animals (data not shown). Although a common finding was represented by the presence of slight-to-moderate lymphoplasmacytic inflammation involving different inner organs, including duodenum, liver, spleen and lungs, no significant association was found between the histological damage and the presence of viral DNA. Furthermore, no lesions were detected in the kidneys of the seven positive animals, findings that may be compatible with the potential role of hedgehogs as a reservoir of this novel virus at least for the hedgehog population assessed in this study.

By comparing the partial VP sequences obtained from the detected viral strains of different tissue origin (data not shown), no changes were observed in the eight available variable regions (VRs) [2], including in the VR-III and VR-VI regions that have been involved in the control of parvovirus tissue tropism [41].

The NS1 sequences displayed high identity (95.8–100% nt) to each other and the highest identity (89.1–97.2% nt) to HhChPV strains previously found in Amur hedgehogs in China in 2018 [22] and in European hedgehogs in Apulia Region, Southern Italy, in 2023 [21]. Identity with the prototype Italian strain was 96.1–97.2% nt. The overall high sequence identity (89.7–99.4% nt) among the hedgehog ChPV strains was also confirmed when reconstructing the nearly full-length genome sequences of four HhChPVs identified in this study. High sequence conservation was observed both in the NS1 (90.5–99.2%) and in the partial VP (89.3–99.4%) encoding genes. Based on NS1 aa sequence phylogenetic analysis, all the hedgehog viral strains grouped tightly (91.5–99.0% aa identity), segregating in a well-defined cluster comprising the most genetically related bat ChPVs (aa identities of 60.3–62.5%) [8,34], and ChPVs were identified in Tasmania devils (61.1–61.4%), rodents (60.5–61.5%), American bleak bears (59.1–59.7%), and non-human primates (59.0–59.6%). Amino acid identities with all other available ChPV strains were lower than 45.6%. Following the ICTV classification criteria, parvoviruses within the same species should have >85% aa NS1 identity, while different viral species within the same genus should have >35% aa identity with an NS1 coverage > 80% [42]. Accordingly, the ChPVs detected in this study should be classified together with the hedgehog strains previously identified in China and Italy as members of a candidate novel species within the genus *Chaphamaparvovirus*, with the proposed name “Chaphamaparvovirus erinaceid 1”.

## 5. Conclusions

In conclusion, the results of this study provided further evidence that ChPV represents a common component of hedgehog virome. Parvoviruses are known to cause a wide spectrum of diseases in humans and animals, such as gastrointestinal illness, immune suppression, and immuno-mediated pathologies. Although corroborating earlier evidence that HhChPV can target intestinal and extraintestinal tissues, our findings warrant further research to decipher the pathogenic roles, if any, of these viruses or their ability to cause persistent infections in hedgehogs as natural animal reservoirs. This could be particularly relevant for species conservation, as viral infections, such as those caused by HhChPV [21], may threaten vulnerable populations of hedgehogs. Furthermore, it is clear that the monitoring and control of viral pathogens in wildlife rescue centers is essential to prevent outbreaks, protect the health of rehabilitated animals, and minimize the risk of introducing pathogens back into natural ecosystems during reintroduction efforts. Finally, since hedgehogs may occasionally come in contact with humans, investigating the hedgehog virome may be relevant not only in terms of animal conservation but also for possible One Health implications.

## Figures and Tables

**Table 1:** A list of oligonucleotides used in this study for the detection and characterization of HhChPVs. Nucleotide position refers to the sequence of the HhChPV strain ITA/2022/hedgehog/265 (GenBank accession no. OQ919797).

Oligonucleotide	Position	Sequence (5′-3′)	Sense	Use	References
2406 F Chap ErEu	1949–1965	GGCGTTTCTGTACCAAAGAGGAA	+	Screening qPCR	[21]
2407 R Chap ErEu	2039–2061	GCATTTGCAGCGATGTTCACTAG	–	Screening qPCR	
316 P Chap ErEu Pb	2012–2037	FAM-TGCATGATACTACCTTTCATTGCAGA-BHQ1	+	Screening qPCR	
2421 HhChPV F	1124–1146	TGGTAGAACAACCAGATCCGACT	+	Screening PCR	This study
2410 HhChPV R	2130–2152	GTTGTTGTACGGGTTGTTCTCCT	–	Screening PCR	
2535 HhChPV F	334–356	CTTCACGACAAGGTGAGGAGGAA	+	Sequencing	
2537 HhChPV R	1321–1294	TGGCTTTTTCTAACTCTTGTTTCTGTCT	–	Sequencing	
2416 HhChPV F	1698–1719	CGCTTTCCTGTCAGGGCTAAAA	+	Sequencing	
2540 HhChPV F	3217–3239	TGCCAATCCACGAAATGTTTCCA	+	Sequencing	
2424 HhChPV R	3458–3431	ACCACTTAGCAATTTGATCAAGATTAAA	–	Sequencing	
2544 HhChPV R	3907–3929	TGGCATACACCTGTCTCCAAGAG	–	Sequencing	

**Table 2:** The quantification of HhChPV DNA in tissues samples available for eight infected hedgehogs.

Hedgehog ID	Collection	Quantity (DNA Copies/g)					
		Duodenum	Liver	Kidney	Spleen	Lung	Brain
#592-2019	A	3.01 × 10^5^	-	-	3.57 × 10^2^	-	-
#618-2019	A	2.98 × 10^5^	-	3.13 × 10^7^	1.39 × 10^4^	-	-
#622-2019	A	-	1.29 × 10^3^	2.72 × 10^3^	-	-	-
#1082-2021	A	-	6.67 × 10^2^	3.08 × 10^5^	7.23 × 10^3^	8.27 × 10^2^	-
#328-2022	A	2.02 × 10^4^	-	4.67 × 10^2^	-	-	-
#403-2022	A	7.87 × 10^2^	-	3.03 × 10^3^	-	-	-
#458-2022	A	8.17 × 10^2^	-	5.03 × 10^4^	-	-	-
#637-2022	B	6.70 × 10^4^	3.73 × 10^5^	1.21 × 10^8^	-	-	-

## Data Availability

The data supporting the findings of this study are openly available in the GenBank database [https://www.ncbi.nlm.nih.gov/genbank/] with accession numbers PQ112546-PQ112560.

## Supplementary Materials

The following supporting information can be downloaded at https://www.mdpi.com/article/10.3390/ani14243624/s1, Table S1: Population characteristics of the molecular-positive European hedgehogs.
