# Supplementary material for: European Hedgehogs as Hosts of Chaphamaparvovirus, Italy
Source: Animals (Basel). 2024 Dec 16;14(24):3624. doi: 10.3390/ani14243624 (PMC11672586; doi:10.3390/ani14243624)
Supplement: Supplementary file 1 [file animals-14-03624-s001.zip › animals-3306496-supplementary.pdf]

Table S1: Population characteristics of the molecular positive European hedgehogs.

Hedgehog ID	Sex	Age	Region of origin	Cause of death	Collection	Date of admission	Date of death
389/18	M	Adult	Piedmont	Infectious/parasitic diseases	A	05/01/2018	05/11/2018
293/22	F	Adult	Piedmont	Unknown	A	11/26/2021	n.a.
328/22	M	Juvenile	Piedmont	Trauma	A	04/15/2021	04/15/2021
403/22	M	Adult	Piedmont	Infectious/parasitic diseases	A	03/18/2022	03/23/2022
413/22	F	Adult	Piedmont	Trauma	A	10/19/2022	10/19/2022
414/22	M	Adult	Piedmont	Trauma	A	09/30/2021	09/30/2021
416/22	F	Adult	Piedmont	Trauma	A	03/28/2022	03/28/2022
458/22	M	Juvenile	Piedmont	Trauma	A	09/04/2021	n.a.
478/22	M	Adult	Piedmont	Trauma	A	04/08/2022	04/08/2022
481/22	F	Adult	Piedmont	Trauma	A	04/10/2022	04/11/2022
484/22	F	Adult	Piedmont	Unknown	A	09/17/2022	11/10/2022
592/19	M	Adult	Piedmont	Infectious/parasitic diseases	A	10/28/2018	11/19/2018
618/19	M	Adult	Piedmont	Trauma	A	05/27/2019	05/27/2019
619/19	M	Adult	Piedmont	Infectious/parasitic diseases	A	05/21/2019	05/22/2019
622/19	M	Juvenile	Piedmont	Infectious/parasitic diseases	A	05/30/2019	06/01/2019
634/22	M	Adult	Piedmont	Trauma	B	05/17/2022	05/17/2022
635/22	M	Adult	Piedmont	Infectious/parasitic diseases	B	n.a.	n.a.
636/22	M	Adult	Piedmont	Trauma	B	n.a.	n.a.
637/22	M	Adult	Piedmont	Trauma	B	n.a.	n.a.
650/22	F	Juvenile	Piedmont	Infectious/parasitic diseases	A	10/05/2021	10/09/2021
656/22	M	Juvenile	Piedmont	Trauma	A	04/17/2021	04/17/2021
701/22	M	Adult	Piedmont	Infectious/parasitic diseases	B	06/04/2022	n.a.
741/22	M	Adult	Piedmont	Unknown	B	06/13/2022	06/14/2022
742/22	M	Adult	Piedmont	Infectious/parasitic diseases	B	06/16/2022	06/18/2022
745/22	F	Juvenile	Piedmont	Infectious/parasitic diseases	B	07/03/2022	07/03/2022
933/22	F	Adult	Piedmont	Trauma	B	07/03/2022	07/04/2022
1038/21	F	Juvenile	Piedmont	Trauma	A	07/17/2021	07/17/2021
1066/21	F	Juvenile	Piedmont	Infectious/parasitic diseases	A	08/02/2021	n.a.
1070/21	F	Adult	Piedmont	Infectious/parasitic diseases	A	07/28/2021	07/29/2021
1071/21	M	Adult	Piedmont	Trauma	A	08/02/2021	08/02/2021
1082/21	M	Juvenile	Piedmont	Trauma	A	02/17/2021	07/17/2021
1123/21	M	Adult	Piedmont	Trauma	A	04/15/2021	04/15/2021
1147/22	F	Adult	Piedmont	Infectious/parasitic diseases	A	08/17/2022	10/13/2022
1148/22	F	Adult	Piedmont	Trauma	A	10/24/2022	10/24/2022
1279/19	F	Juvenile	Piedmont	Infectious/parasitic diseases	A	06/01/2019	07/01/2019
1785/22	M	Juvenile	Piedmont	Trauma	B	11/08/2022	11/12/2022

7/21	M	Juvenile	Aosta Valley	Trauma	C	n.a.	n.a.
11/21	M	Adult	Aosta Valley	Trauma	C	n.a.	n.a.
